# The HEPS project

**DOI:** 10.1107/S1600577518012110

**Published:** 2018-09-26

**Authors:** Yi Jiao, Gang Xu, Xiao-Hao Cui, Zhe Duan, Yuan-Yuan Guo, Ping He, Da-Heng Ji, Jing-Yi Li, Xiao-Yu Li, Cai Meng, Yue-Mei Peng, Sai-Ke Tian, Jiu-Qing Wang, Na Wang, Yuan-Yuan Wei, Hai-Sheng Xu, Fang Yan, Cheng-Hui Yu, Ya-Liang Zhao, Qing Qin

**Affiliations:** a Key Laboratory of Particle Acceleration Physics and Technology, Institute of High Energy Physics, Chinese Academy of Sciences, Beijing, People’s Republic of China

**Keywords:** High Energy Photon Source (HEPS), diffraction-limited storage ring, modified hybrid seven-bend achromat

## Abstract

The High Energy Photon Source (HEPS), a 6 GeV green-field diffraction-limited storage ring light source, will be built in Beijing, China. The HEPS design has been evolving for about ten years, and is now mostly finished and ready for construction. The storage ring is based on a modified hybrid seven-bend achromat (7BA) design, where bending magnets with reverse bending angles and longitudinal gradients are adopted to reach an ultralow natural emittance of 34.2 pm with a circumference of 1360.4 m. The central slice of the dipole in the middle of the modified hybrid 7BA, with flexible magnetic field, is used as the source of the bending-magnet beamline. Moreover, alternating high- and low-beta sections are specially designed to generate and deliver X-ray synchrotron radiation with high brightness of 5 × 10^22^ photons s^−1^ mm^−2^ mrad^−2^ (0.1% bandwidth)^−1^. Here, the HEPS storage ring design and solutions to the challenges inherent in this ultralow-emittance design are presented.

## Abstract 2

Details of the High Energy Photon Source (HEPS), a 6 GeV green-field diffraction-limited storage ring light source to be built in China, are presented.

## 1. Introduction   

The High Energy Photon Source (HEPS) is the first high-energy diffraction-limited storage ring (DLSR) light source to be built in China. In early 2008 it was proposed to build a high-energy kilometre-scale ring-based light source with a double-bend achromat (DBA) design, to meet the increasing demands for high-brightness hard X-ray synchrotron radiation in China (Jiang *et al.*, 2014 ▸). Since then, the accelerator design has been continuously evolving, keeping up with the advances in accelerator physics and technology around the world towards the so-called diffraction-limited storage ring (DLSR) light sources with multi-bend achromat (MBA) lattice. It was in 2012 that a standard seven-bend achromat (7BA) design with a natural emittance of 75 pm at 5 GeV was first proposed for HEPS (Xu & Jiao, 2013 ▸), and, after that, different MBA designs were investigated to explore the ultimate performance of HEPS (Jiao & Xu, 2015 ▸; Xu *et al.*, 2016*a* ▸; Jiao, 2016 ▸; Jiao *et al.*, 2017*a* ▸; Peng *et al.*, 2017 ▸). In 2014, after iterative discussions among accelerator and beamline experts, the design goal of HEPS was clarified: to construct a 6 GeV DLSR light source with a circumference of ∼1300 m. To develop the key hardware techniques required for constructing a DLSR light source and obtain an optimal lattice design for HEPS, an R&D project called the High Energy Photon Source Test Facility (HEPS-TF) was launched in 2016. The HEPS-TF project is now almost completed. The key technologies have been demonstrated, *e.g.* high-gradient quadrupoles, small-aperture vacuum chamber with non-evaporable getter coating, and high-precision power supplies. The physics design of the HEPS has been mostly finished (Jiao *et al.*, 2018*a* ▸; Xu *et al.*, 2018 ▸). After being officially approved, construction of HEPS will begin by the end of 2018.

**Figure 1:**
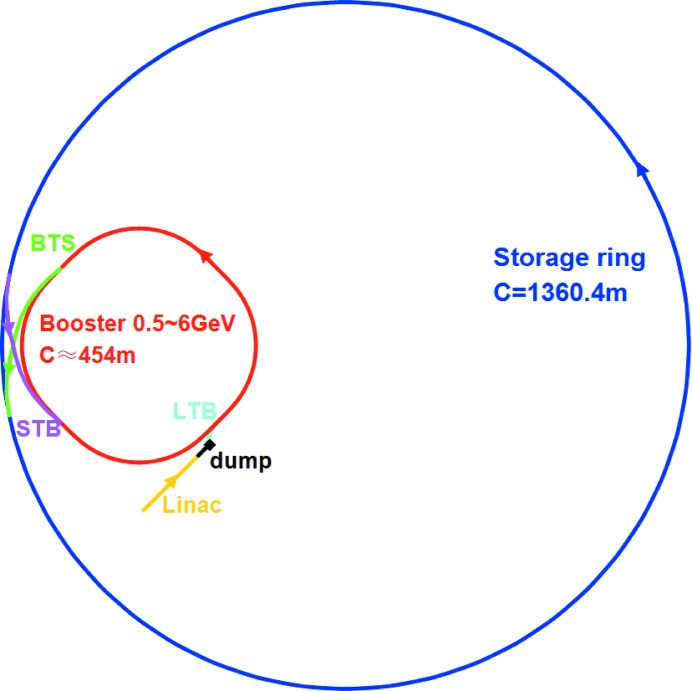
Schematic layout of the HEPS project

**Figure 2:**
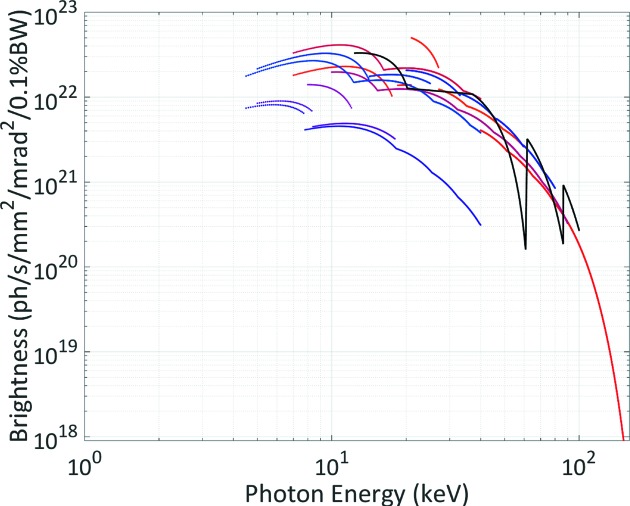
The available spectral brightness for HEPS operated in the high-brightness mode, evaluated by taking the intrabeam scattering effect, impedance and harmonic cavity into account.

As shown in Fig. 1 ▸, HEPS consists of a 500 MeV linac, a booster that ramps beam energy up to 6 GeV, and a storage ring. There are three transport lines connecting the linac, booster and the storage ring. In the first construction phase, 14 beamlines will be built. The parameters of the insertion devices (IDs) for these 14 beamlines have been optimized according to user requirements. Two operating modes, *i.e.* high-brightness mode (200 mA, 680 bunches) and high-bunch-charge mode (200 mA, 63 bunches), are considered. The available spectral brightness of the high-brightness mode is shown in Fig. 2 ▸. A brightness of 5 × 10^22^ photons s^−1^ mm^−2^ mrad^−2^ (0.1% bandwidth)^−1^ is expected at the photon energy of 21 keV for HEPS. In the following sections we will describe the storage ring lattice design and optimization, optics correction, injection, collective effects and the injector design.

## 2. Storage ring design   

**Figure 3:**
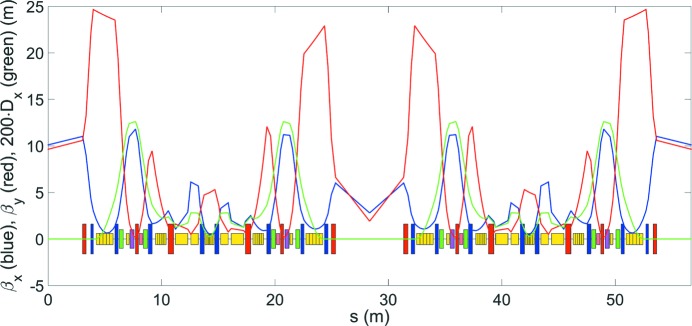
Optical functions and layout of one super-period of the HEPS storage ring. Each super-period comprises two modified hybrid 7BAs.

The HEPS storage ring comprises 48 7BA cells that are grouped in 24 super-periods, with a circumference of 1360.4 m and a natural emittance of 34.2 pm. The layout and optical functions of one super-period are shown in Fig. 3 ▸, and the main parameters of the storage ring are summarized in Table 1 ▸.

In the HEPS 7BA lattice design, we basically followed the so-called hybrid MBA concept (Farvacque *et al.*, 2013 ▸), that was first proposed for the ESRF-EBS project. This novel lattice structure can overcome the difficulties faced in a high-energy DLSR design with a standard MBA lattice, where the sextupole strengths required for chromatic correction increase significantly as the natural emittance is continuously reduced towards a few tens of picometres. Taking the hybrid 7BA (hereafter referred to as H-7BA) as an example, it has three TME (theoretical minimum emittance)-like cells in the middle and two DBA-like cells on two sides. Dispersion bumps are created in each DBA-like cell with chromatic sextupoles located therein, such that the sextupole strengths can be kept at an acceptable level that can be reached using conventional magnet technology. This comes at a price, that the Courant–Snyder parameters in the DBA-like cells are not very close to the TME conditions (Teng, 1984 ▸), leading to a comparably low emittance reduction efficiency. Thus, in order to achieve a minimized emittance as well as a compact layout, measures including bending magnets combined with longitudinal gradients (BLGs) and bending magnets combined with horizontally defocusing gradients (BDs) and dedicated focusing quadrupoles (QFs) with higher gradients than in the standard 7BA cell are used in the DBA-like cells and the middle TME-like cells, respectively. Furthermore, the phase advance between each pair of sextupoles is matched to be close to (if not exactly at) 3π in the horizontal plane and π in the vertical (*i.e.* the −I transportation) and octupoles may also be used, to minimize the nonlinear driving terms induced by sextupoles and greatly facilitate subsequent nonlinear optimization. Studies (Jiao *et al.*, 2017*b* ▸) showed that if 48 identical H-7BAs are used, the natural emittance of the HEPS storage ring can be pushed down to about 45 pm, while keeping a sufficiently large dynamic aperture (DA) for on-axis injection.

To further improve the performance, several modifications were proposed based on the H-7BA (Jiao *et al.*, 2018*a* ▸). For simplicity, in the following we refer to the modified H-7BA as MH-7BA. In the first place, the middle cell of an H-7BA was replaced by a novel cell very similar to that first proposed (Streun & Wrulich, 2015 ▸) for the SLS-2 project. In this novel cell, the dipole is a BLG instead of a BD, with the highest field in its central slice; neighboring the dipole, there are two dedicated defocusing quadrupoles (QDs) and two anti-bends combined with focusing gradients (*i.e.* QFs horizontally displaced outward from the ring by a few millimetres). A performance comparison of different cells has been performed (Jiao *et al.*, 2018*b* ▸) based on the HEPS design parameters. The results indicated that the former cell (the middle cell of an H-7BA) promises an emittance of about two times that of the TME, and close to the TME if replacing the QFs with anti-bends as was done for the Advanced Photon Source Upgrade design (Borland *et al.*, 2016 ▸), while the latter (the middle cell of a MH-7BA) allows for an emittance 30–50% lower than the TME as long as the cell is long enough, *i.e.* not less than 2.6 m. Secondly, unlike the case in an H-7BA where the main dipoles are comparably weak and consequently additional bending magnet (BM) sources are required, in the middle cell of a MH-7BA the central slice of the LGB itself can serve as a BM source. Furthermore, as long as the length and total bending angle of the LGB are kept the same, the LGB peak field can be changed according to different user requirements, generating flux with different critical photon energy while causing little perturbation to beam dynamics. Thirdly, one family of QFs within the dispersion bump was replaced by anti-bends. This promises more flexible control of the dispersion functions and helps to achieve an even lower emittance. Fourthly, different from an H-7BA which generally has symmetric optics, the optics of an MH-7BA is matched to create high- and low-beta sections on different sides of the 7BA. One can achieve as high as possible brightness in the low-beta straight sections by reducing the beta functions to be close to those for optimal matching of the electron and photon beam, and, on the other hand, obtain adequate DA in the high-beta straight sections to ensure high injection efficiency.

Experiences suggested that the parameter adjustment space for an optimal performance is quite limited in a DLSR design and the nonlinear dynamics in a DLSR are more coupled with the linear optics compared with a third-generation light source. Furthermore, it is difficult (if not impossible) to find one or a few factors that are effective in optimizing the DA and momentum acceptance (MA) (Jiao & Xu, 2018 ▸). Thus, in the HEPS design we performed a global optimization with a rational combination of the PSO and MOGA algorithms, which has been demonstrated to be more effective than either of them alone in approaching the true global optima of a typical explorative multi-objective problem with many local optima (Jiao & Xu, 2016*a* ▸,*b* ▸, 2017 ▸).

In optimization, all tunable parameters (more than 60 parameters) were varied; as many constraints and limitations as possible were considered. Two optimizing objectives were used to characterize the overall performance of a lattice: one is the weighted brightness and the other is the weighted DA, which is actually the product of DA and MA obtained from numerical tracking and with some normalization treatment.

Through optimization it was empirically found that the nonlinear performance is more sensitive to the horizontal phase advance between the sextupole pair than to the vertical. When relaxing the limitation on the vertical phase advance, a stronger vertical focusing than usual was suggested for higher brightness and larger DA. For the latest HEPS design, the nominal tunes are (114.14, 106.23). This design results in adequate DA and local MA for on-axis injection and a reasonable lifetime, even when the scenario of a realistic machine is considered. Details will be discussed in the next section.

## 3. Optics correction   

To achieve an ultralow emittance, much stronger focusing is required in a DLSR design than in the third-generation light sources. The state-of-the-art design of quadrupoles for HEPS has a high gradient, up to 80 T m^−1^. This, in turn, means that the orbit distortion is very sensitive to the transverse mis­alignments of quadrupoles. In fact, it was found that a dedicated first-turn-around strategy is mandatory to find the closed orbit (Zhao *et al.*, 2017 ▸). In order to keep the closed orbit distortion after correction at a small enough level, the alignment of magnets on the same girder and between adjacent girders are required to be better than 30 µm r.m.s. and 50 µm r.m.s., respectively, and 576 beam position monitors and 480 orbit correctors along the ring are used for trajectory and closed-orbit distortion corrections. In addition, to control the effects of the dynamic errors (*e.g.* ground vibration and power supply ripples) and maintain a beam orbit stability of better than 10% of r.m.s. beam sizes within the radiation sources, a fast orbit feedback system using all the beam position monitors operated in 22 kHz fast-acquisition mode and 192 fast correctors out of the 480 correctors is under design.

Furthermore, the strong focusing causes large natural chromaticities, which necessitates strong sextupoles to correct the linear chromaticity to large enough positive values, *e.g.* (+5, +5). Simulation studies showed that, after orbit correction, the offset of the sextupoles center relative to the beam orbit is about 70 µm r.m.s., leading to a strong feed-down effect and forming the dominating contribution to the optics distortion. This effect, if it is not well controlled, will deteriorate the beam dynamics and lead to an evident beam emittance growth and a substantial DA reduction. To this end, a novel scheme to locally correct the sextupole feed-down effects has been developed (Duan *et al.*, 2018*b* ▸; Ji *et al.*, 2018 ▸). We proposed to install dedicated movers for sextupoles such that the sextupole transverse positions can be remotely adjusted with a high precision of 5 µm r.m.s. The sextupole offsets are used in the LOCO fitting (Safranek, 1997 ▸), and then the sextupole movers are adjusted according to the fitting results. After several iterations of such a correction process, it is feasible to reach a very good correction of the linear optics and a substantial recovery of the DA. The r.m.s. residual beta beating is about 0.5% in both horizontal and vertical planes, and the r.m.s. deviation of horizontal dispersion is about 0.7 mm. The horizontal emittance growth is smaller than 10% for most of the error settings, and the vertical emittance is typically at the picometre level. In routine operation, it is probably necessary to adjust the vertical emittance to reach a balance between high brightness and long beam lifetime. To this end, three skew quadrupole correction coils are used in each 7BA, with two of them in the dispersive region and the other one in the dispersion-free region, to enable a fine tuning of the vertical emittance.

The ID effects were also considered. They cause an additional energy loss per turn of about 1.5 MeV and a reduced natural emittance of 27.5 pm at zero-current approximation, and induce (if not corrected) a vertical tune shift of about 0.03. Studies ensured that the tune shift caused by each ID can be corrected with nearby quadrupoles, and the beam orbit distortion caused by ID integral field errors can be locally corrected with four correctors near the ID (Li *et al.*, 2017 ▸).

**Figure 4:**
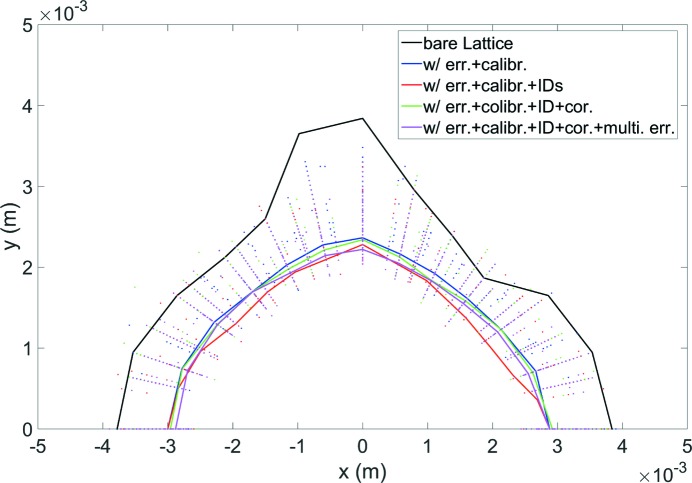
DA of the HEPS storage ring, for the bare lattice (black) and for the case with practical errors at different steps of the correction procedure (colored dots). The colored curve represents the 20th-percentile smallest DA among the random error seeds.

The DA at the center of the high-beta straight section and the LMA of the bare lattice at each step of the lattice calibration were evaluated, where the multipole error effects were also considered. The results are shown in Figs. 4 ▸ and 5 ▸. The DA after correction is about 2 mm in the vertical plane and 3 mm in the horizontal plane, meeting the requirements of on-axis injection to the storage ring, which will be discussed next.

## 4. Injection   

In the past few years, different injection schemes have been studied for HEPS, including off-axis injection with a pulsed sextupole kicker (Jiao & Xu, 2013 ▸), two novel longitudinal injection schemes (Xu *et al.*, 2016*b* ▸; Duan *et al.*, 2016 ▸; Jiang *et al.*, 2018 ▸) as well as the current baseline injection scheme, *i.e.* on-axis swap-out injection (Emery & Borland, 2003 ▸). The swap-out injection scheme is chosen based on the following considerations. First of all, on-axis swap-out injection requires a much smaller DA compared with off-axis injection, and it potentially enables a further reduction of the emittance and an enhancement of the brightness. Second, bunch-by-bunch swap-out injection is expected to be more transparent to the user experiments than off-axis injection. Last but not least, both the swap-out and longitudinal injection schemes require an ultrafast kicker system, while the swap-out injection is less technically demanding in terms of the much relaxed requirement on the pulse width of the kicker system in contrast to the longitudinal injection schemes.

**Figure 6:**
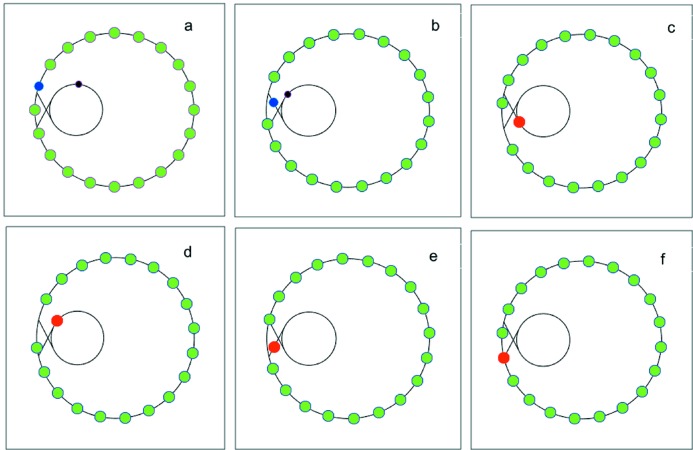
The whole process of filling a bunch in a storage ring.

The major challenge for the swap-out injection is to deliver full-charge bunches to the storage ring, in particular to meet the need of the high-bunch-charge mode with 14.4 nC per bunch. To this end, we proposed a high-energy accumulation scheme (Duan *et al.*, 2018*a* ▸) where the booster is used as an accumulator ring at 6 GeV. As shown in Fig. 6 ▸, one electron bunch that loses a fraction (*e.g.* 10%) of charge (colored blue) is extracted from the storage ring (*a*), injected to the booster after passing through a transport line (*b*), merged with one bunch of the booster (colored black) that has been injected from the linac and ramped up to 6 GeV (*c*); after about 10000 turns in the booster, this bunch (colored red) is extracted from the booster (*d*), and then re-injected into the storage ring (*f*) after passing through another transport line (*e*). In this way, the booster needs only to store and accelerate bunches with a moderate charge, which can overcome the difficulty of storing a high-charge bunch in the booster due to single-bunch instability that is particularly strong near the injection energy.

**Figure 7:**
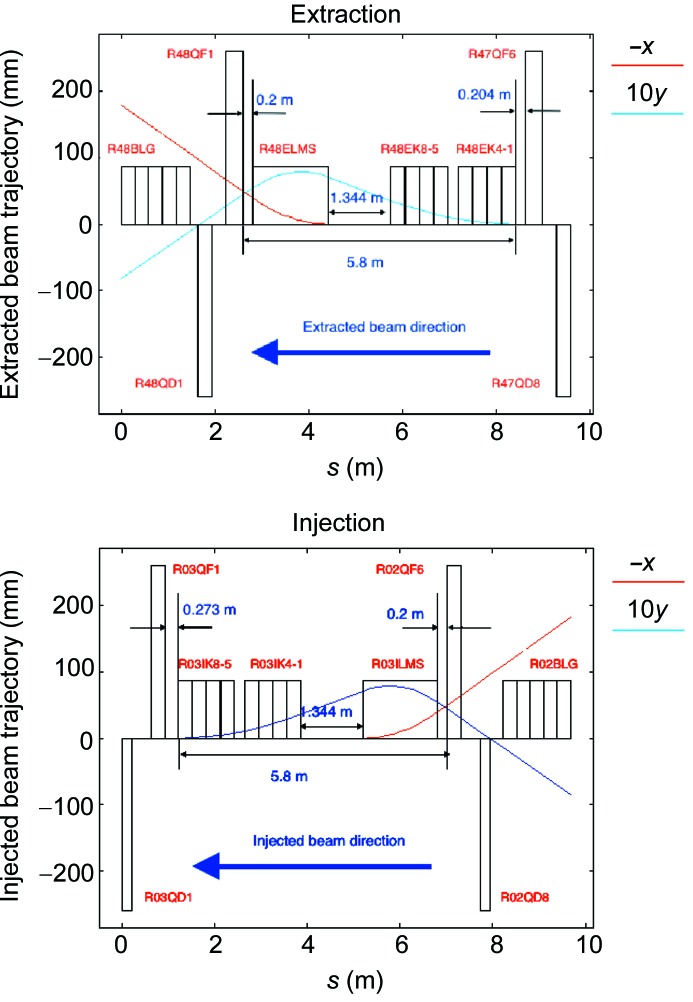
Layout of the injection and extraction regions of the HEPS storage ring.

The minimum bunch spacing is 6 ns in the storage ring. To enable swap-out injection and extraction in a bunch-by-bunch manner in the storage ring, both the injection and extraction kickers are designed to have a very short rise time, flat-top and fall time. Based on the R&D experiences in the HEPS-TF project, we chose to use eight stripline kickers of 300 mm in both the injection and extraction regions, and design the associated high-voltage pulsers such that they provide a Gaussian-shape pulse with a FWHM of about 4.5 ns. In addition, Lambertson magnets tilted in yaw, roll and pitch (Abliz *et al.*, 2018 ▸) were employed to separate the stored beam from the injected or extracted beam. The layouts of the injection and extraction regions are shown in Fig. 7 ▸. For the injection and extraction systems of the booster, kickers and Lambertson magnets based on conventional technologies will be adopted, reducing the technical challenges as much as possible. Based on the latest design of the storage ring and the injector (details can be found in the *Injector design* section[Sec sec6]), preliminary simulation studies showed satisfactory transmission efficiency in the whole injection process.

## 5. Collective effects and beam lifetime   

To realize the strong focusing for minimizing beam emittance, small aperture magnet and vacuum systems were adopted in the HEPS design. The inner radius of the vacuum chamber in the storage ring is mostly 11 mm, which induces much stronger impedance compared with the third-generation light sources. The strong impedance, together with the high bunch density and small momentum compaction factor of the HEPS storage ring, makes the collective effects, including the beam instabilities, intra-beam scattering (IBS) and Touschek effects, more significant.

The longitudinal and transverse impedances of various vacuum components have been evaluated piecewise (Wang *et al.*, 2017*b* ▸). The resistive wall impedance was calculated with analytical formulae (Wang & Qin, 2007 ▸), and the geometrical impedances were calculated numerically with *ABCI* and *CST*. An impedance budget including all these elements has been obtained. This allows a global view of the contribution of different components to the total impedance budget, providing hints for further optimization of critical vacuum components. Longitudinal impedance measurements on the prototypes of specific devices have been performed using the wire method (Tian *et al.*, 2018*a* ▸), as a benchmark for the analytical and numerical estimations.

In order to lower the particle intensity and mitigate the IBS and Touschek effects, third-harmonic cavities were proposed. The IBS inflated beam parameters are listed in Table 2 ▸, where the influence of the longitudinal single-bunch dynamics with impedance and harmonic cavities has been included.

Single-bunch and multi-bunch instabilities have been evaluated based on the impedance budget (Duan *et al.*, 2017 ▸; Xu & Wang, 2018 ▸; Wang *et al.*, 2017*a* ▸). The most important single-bunch instabilities that affect the beam quality are the microwave instability in the longitudinal plane and transverse mode coupling instability (TMCI) in the transverse plane. The threshold bunch charges for the single-bunch instabilities are listed in Table 3 ▸. One can see that the harmonic cavities are helpful in increasing the threshold. For the TMCI instability, a large enough positive chromaticity is essentially required to suppress the instability and keep beam stable at 200 mA. Note that when the storage ring operated in high-brightness mode, the bunch charge (14.4 nC) is much larger than the threshold of the microwave instability, which will not cause beam loss but an evident increase in the r.m.s. energy spread, affecting the quality of the photon beam. The potential influence to user experiments and possible solutions are under study. On the other hand, the available highest beam current is mainly determined by the multi-bunch instabilities induced by the transverse resistive wall impedance and high-order modes (HOMs) of the RF cavities, whatever the operation mode of the storage ring is. The transverse resistive wall instability is induced by the resonance at zero frequency of the resistive wall impedance. For HEPS the growth time of the most dangerous instability mode is about 0.5 ms. A bunch-by-bunch transverse feedback system will be used to cure this instability. The instabilities induced by HOMs of the geometrical impedances, including those of the RF cavities, were investigated. It was found that the HOMs of the 166.6 MHz RF cavities, if not controlled, can cause instabilities with growth time beyond the ability of the state-of-the-art feedback system. Now a HOM damper is under design and optimization for the RF cavities in order to reduce the shunt impedance to a sufficiently low level.

Associated with the small transverse beam size and high beam intensity, beam ion instability may be excited by the residual gas accumulated in the potential well of the electron beam and affect the machine performance. Both analytical estimations and numerical simulations were performed (Wang *et al.*, 2018 ▸; Tian *et al.*, 2018*b* ▸). Studies showed that the growth time is 2 to 4 ms, which can be cured with the transverse feedback system.

The beam lifetime is mainly dominated by the Touschek lifetime in the ultralow-emittance rings. Based on the tenth-percentile smallest LMA among random error seeds, the Touscheck lifetime was estimated to be 4.0 h for the high-brightness mode and 0.9 h for the high-bunch-charge mode. The vacuum lifetimes due to elastic gas scattering and gas bremsstrahlung, assuming a vacuum pressure of 1 nTorr (with 80% H_2_ and 20% CO), were estimated to be 136.7 and 257.8 h, respectively. It is expected to have a beam lifetime of 3.8 h for the high-brightness mode and 0.8 h for the high-bunch-charge mode. To keep high brightness during operation, top-up injection is planned, with the electron beam refilled every 15 to 20 s.

## 6. Injector design   

The HEPS injector consists of a linac, a low-energy transport line, a booster synchrotron and two high-energy transport lines. The booster is located in a tunnel separated from the storage ring so as to significantly reduce the impact of the booster ramping on the operation of the storage ring.

In order to achieve a high stability in operation, we chose to use mature technologies in the injector design wherever possible (Li *et al.*, 2018 ▸). The linac uses a thermionic gun and S-band normal conducting accelerating tubes to generate an electron pulse with a charge of up to 4 nC and accelerate it to 500 MeV. The booster design is based on a fourfold symmetric FODO lattice (Peng *et al.*, 2018 ▸), with a natural emittance of 33 nm at 6 GeV, and an equilibrium r.m.s. energy spread of 9.6 × 10^−4^. The repetition rate of the booster was selected as 1 Hz. It is required that the booster can provide electron beam with up to ten bunches and 2 nC in each bunch to the storage ring.

As mentioned, the booster at 6 GeV is also used as an accumulator ring. The nonlinear beam dynamics of the booster have been optimized, and the high-energy injection and extraction systems of the booster have been designed and optimized, to ensure good capture efficiency. Two high-energy transport lines were designed to connect the booster and the storage ring. Their lengths were carefully adjusted to ensure the bunch extracted from the storage ring can return to the same bucket when it is re-injected to the ring after replenishing charge in the booster.

## 7. Conclusions   

The HEPS will be the first high-energy DLSR light source in China. Up to now, the storage ring has been designed based on a modified hybrid 7BA lattice. The related physics issues, including optics correction, injection design, collective effects and injector design, have been carefully studied. In the present HEPS design as many features as possible are adopted to maximize the brightness, ensuring that, once built, the HEPS will be one of the most brilliant light sources in the world.

On the other hand, this design still leaves room for further improvement of the performance. For example, the highest quadrupole gradient used in the current design is 80 T m^−1^, which can be increased to a higher value to achieve lower emittance and even higher brightness. In the present injection design we reserved the compatibility of longitudinal injection to a large extent which in the future will allow beam experiments to be carried out to verify the longitudinal injection on a DLSR.

Finally, to realize the unprecedented high-quality design performance, successful engineering design and implementation are essential. Fortunately, most of the required cutting-edge technologies have been demonstrated in the HEPS-TF project, providing a solid basis for the HEPS project, whose construction will be started by the end of this year.

## Figures and Tables

**Figure 5:**
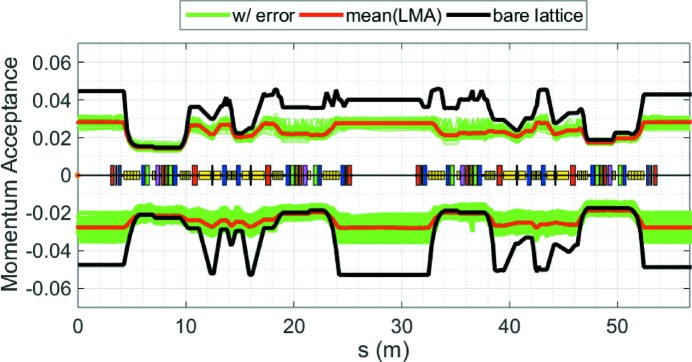
Local momentum acceptances (LMAs) of one super-period of the HEPS storage ring, for the case with only the bare lattice (black) and with practical errors (blue curves represent results with different error seeds and red represents the average LMA).

**Table 1:** Main parameters of the HEPS storage ring (bare lattice)

Parameters	Values	Units
Beam energy *E*_0_	6	GeV
Beam current *I*_0_	200	mA
Circumference	1360.4	m
Horizontal damping partition number *J*_*x*_/*J*_*y*_/*J*_*z*_	1.85/1/1.15	–
Natural emittance	34.2	pm rad
Working point (*x*/*y*)	114.14/106.23	–
Natural chromaticity (*x*/*y*)	−215/−292	–
Corrected chromaticity (*x*/*y*)	+5/+5	–
Number / length of high-beta straight sections	24 / 6.073	– / m
Beta functions at the center of low-beta straight sections (*x*/*y*)	10.12/9.64	m
Number / length of low-beta straight sections	24 / 6.004	– / m
Beta functions at the center of low-beta straight sections (*x*/*y*)	2.80 / 1.91	m
Momentum compaction	1.56 × 10^−5^	–
Damping time (*x*/*y*/*z*)	10.2 / 18.9 / 16.4	ms
Energy loss per turn *U*_0_	2.89	MeV
Energy spread σ_δ_	1.06 × 10^−3^	–
Fundamental frequency (166.6 MHz) RF voltage	3.64	MV
Harmonic (499.8 MHz) RF voltage	0.65	MV
Bunch length without / with harmonic cavities	4.9 / 29.0	mm
Harmonic number	756	–

**Table 2:** Equilibrium beam parameters with the IBS effect

Parameters	High brightness	High bunch charge
∊_*x*_ (pm)	27.5	33.0
σ_δ_ × 10^−3^	1.1	1.9
σ_*z*_ (mm)	32.0	48.0

**Table 3:** Threshold bunch intensity for the single-bunch instabilities

	Threhold without harmonic cavities (nC)	Threshold with harmonic cavities (nC)
Microwave instability	0.9	2.2
TMCI (ξ = 0)	0.3	0.4
TMCI (ξ = 5)	>30	>30
